# Dental Quacks

**Published:** 1858-12

**Authors:** 


### "DENTAL QUACKS."—*Continued.*

> "Myself I throw, dread Sovereign, at thy feet;
> My life thou shalt command, but not my shame;
> The one my duty owes; but my fair name
> (Despite of death, that lives upon my grave),
> To dark dishonor's use thou shalt not have,
> I am disgraced, impeached, and baffled here;
> Pierced to the soul with Slander's venomed spear;
> The which no balm can cure,—but his heart-blood
> Which breathes this poison."—*Shakspeare.*

MESSRS. EDITORS: Permit me to introduce myself to you and the readers of the *Register*, as the "Dental Quack," the "twin brother" to another "now on the black-list," as well as one among the "villains," "rascals," and dishonorers of their alma mater, referred to in the article on "Dental Quacks," in the last number of the *Register*, and designated "Dr. C." You will please not, however, consider this introduction as an acknowledgment, on my part, that *I really am* said "Dental Quack," etc. Neither need you consider it as a preliminary step to exonerate myself from the charge,—(as I have every reason to believe I will be saved this trouble by the honorable editor of "a country newspaper," through whose inadvertency an opportunity was afforded for it); but you may consider it as opening the way for the expression of a few

> "Thoughts that have tarried in my mind,"

Since the appearance of the article under consideration, in regard to the malicious spirit which undoubtedly prompted, at least, that portion of it referring to "Dr. C.;" and to the effect on the profession, of making the Dental press, under the very propitious garb of "guarding our dental colleges, the medium of gratifying a private malicious feeling, especially on the part of an old and established practitioner, toward one who has but made his *first* advertisement to the world.

I am free to acknowledge, here, that the prosecution of my intention, as intimated, is fraught with several embarassments. (In consideration of which, I would feign ignore the injustice done myself; but a demand, on the part of friends, for justice, and the hope to benefit the profession, by cautioning it against sounding false alarms, will not allow me so to do.) Namely: 1st, The article, with a few *decided exceptions*, *reads* well enough, and its tendency seems to be that of "guarding our dental colleges." 2d, The age and standing of the writer demands *my* deep respect. And, 3d, *He was my own preceptor.* (In another point of view, these considerations *aggravate* the matter.)

In proof of the assertion, that a malicious feeling prompted the charge of quackery against me, I shall embrace the privilege afforded by our common law, and appeal to my past character; and having proved that *good*, by the *same* evidence shall show that the originator of this charge knew my character, *and that had he not been predisposed to injure me*, would neither have supposed me guilty, nor *exposed* my guilt *without a previous warning voice*, had he been satisfied of it. Before presenting this proof, however, it will be necessary to state a few facts explanatory of it.

At a certain stage of my precious (?) and inglorious (?) dental career, it was my lot to meet with, in Cincinnati, and form the acquaintance of Dr. H. R. (which, *as far as it goes*, is the initials of him, who, while he professes to "protect the innocent, and *love the profession*, eagerly grasps, and hurls before the public, a fact, in a manner not only well calculated to brand, as an infamous *dental Quack*, one whose "first entrance into the profession, was to attend the lectures at the Ohio Dental College, during the session of 1856–7; but equally well calculated to stifle in the heart what little aspirations,—peradventure he may have had to attain to the full stature of a *man* in the profession!

Dr. H. R. proposed to me to accompany him to his home, in a neighboring State, for the purpose of pursuing my studies with him. The suggestion meeting my approval, I accompanied him thither. There being no article of agreement between us, it was tacitly agreed, that I should bear my own expenses, and remain with him as long as it was agreeable to those concerned. I had been with him but a short time, when he changed his place of business, leaving me with his brother, at the old stand. Becoming dissatisfied with the amount of practice I was receiving, in compensation for my expenses, I expressed a desire to quit the office to Dr. H. R., by letter; and received, in answer, a letter, from which I make but sufficient extracts to prove an honorable release:

"..........., July 29th, '57.

"FRIEND.......: Your letter of the 27th is just now before me, and I hasten to reply. You seem dissatisfied with ....., and for the reasons you state, feel that it would be better for you to leave. I am sorry that the circumstances are such, that we could not offer such inducements as would be satisfactory to you to remain; but I fear they will not be so, soon, enough to meet your necessities. I can not advise, further than that you consult your interests in the matter, entirely. Wherever you go, you will have my good wishes for your welfare and prosperity."

(I pray thee, doctor, *withhold* thy "good wishes" for my "welfare and prosperity," *if, to express them,* "more stringent means may be called into action," than "the gentle hint" you have already given me by way of paternal chastisement.)

I am now ready to prove the maliciousness of the feelings which prompted the charge made against me; and I deem it most expedient to do this simply by giving Dr. H. R.'s expressed *friendly* feelings toward me,—by calling attention to the relation of preceptor and student as it existed between us!—*and by claiming that it was utterly incompatible with this state of things, that such a charge should be made,*—and that, too, without the least effort on the part of the maker, to ascertain whether or not I was a *quack at heart.*

> "Give me *good* proofs of what you have alleged.
> 'T is not enough to say in such a bush,—
> There lies a thief,—in such a cave, a beast."

Dr. H. R.'s expressed friendly feelings toward me, may be found in the following quotations, which I make from a letter, written by him to myself. (The quotations will also serve as the premised proof of my previous good character, etc.):

"..........., July 5th, '57.

"FRIEND .......: Your very welcome letter reached me in due time. You speak of my friendship for you, and of the value you place on my advice, etc. To which I would say, that, with the acquaintance which I had with you, while here, I formed a very high estimate of your moral and intellectual worth; with your decision of character, and invariable gentlemanly bearing, not only toward me, but toward all with whom you came in contact. And, to your further credit I will say, that as yet I have had no cause to think otherwise. To such a character, it is no way strange that I profess my friendship and assistance."

(I pray thee, doctor, *withhold* thy "friendship" and "assistance," *if, to express them,* "more stringent means may be called into action," than the "gentle hint" you have already given me, by way of *friendly assistance.*)

The charge *not being compatible,* therefore, with *these* feelings, *certainly* is *only compatible with the opposite.*

It remains now, for me to briefly state some of the *effects on the profession* of this kind "love" for it, *thus* expressed. The philosophy (!) used in doing this shall be of no doubtful sort, but emphatically *inductive,* myself being the subject of experiment, I have already intimated a prominent effect of this *quackery* on the profession, namely, its tendency to

*effectually smother*, in its victims, any ambition they may possess to rise in, or benefit it,—by sapping the foundation of character;—

> ..................."That away,
> Men are but guilded loam, or painted clay."

It is unnecessary to say the Dental profession is in its infancy; and that it becomes all "lovers" of it, to fain receive, and not hastily judge, such material proffered for its upbuilding.

Will Dr. H. R. contend that his palliation, of designating me "Dr. C." instead of Dr. D., after following me, *step* by *step*, since my "first entrance into the profession," (in which "following," *he* has narrowly escaped, if at all, catching the quack disease), militates against my assertion, that his charge tends to sap the foundation of my character? If so, I fear he "hasn't traveled" as much as his article; and some of my dental friends, who readily recognize Dr. C., and as readily made known the *joke* to those, whom *most* have I desired to know me, as Cæsar's wife was known, to be not only innocent, *but above suspicion.*

An other effect of such a hasty "reading out" of the profession, is the subversion of the true intent of the Dental press, which is to *build up* the profession; and this, too, not by an eager disposition to *tear down individual members*, even though they be guilty of fraud, *if* "these circulars reach only the profession, while those who are to be most effected are still ignorant of the fraud practiced upon them." Besides, the valuable space taken up by these charges and defenses, is poorly compensated for, at best.

Again,—the *reflex action* of this malicious gratification, *is evil*, *only evil*, and that continually.

Once more, but for a moment, and I submit the question: In all candor I will anticipate the only answer, to me conceivable, which Dr. H. R. can make to the *worse* than *dental quack* charge which I make against him, namely, "In an editorial notice, in a country newspaper, of a copartnership of Dr. C. with Dr. I. Dr. C. is represented as having had *more* practice than he *really had; this error was never corrected. Therefore*, it seems to be my special province, as Dr. C. was my student, and as I have *expressed* my *good wishes* for his *welfare* and *prosperity*, and as I have *promised* my *friendship* and *assistance*, to give him a *gentle* hint, *secretly*, by way of reformation." I will make but one apology for the strength of the doctor's answer, and abide by the decision of those, who, having read both charge and defense, have all the facts in the matter, namely: The offending "editorial notice" was unsolicited by me; *was not an advertisement.* The statement having been made by the editor, without my knowledge or solicitation, I felt no blame resting upon me for it; nor did it strike me as a duty to demand the editor to make the very awkward acknowledgment, that he had extended weeks of time into years, and made a tyro into a skillful operator, in his notice of me; but I considered it my duty to give the facts in the matter, when questioned upon it, which I always did. Yours, truly, Dr. C. C. D.

—

............, November 15, 1858.

Drs. Taft & Watt: Having seen an article in the *Dental Register* for September, headed "Dental Quacks," in which a worthy member of the dental profession is severely censured, for publishing, as the writer alleges, a Card containing false statements, for the purpose of securing business,—I beg the privilege of making an explanation of the matter, which will, I trust, wholly exonerate the gentleman thus charged, from all blame. The "Card" alluded to by the writer in the *Register*, was simply an editorial notice, written by myself, without consultation or even acquaintance at that time, with the "Dr. C." referred to. The error, in stating that "Dr. C." had the advantage of "eight years' experience," was entirely my own, and resulted from a hasty reading of some dim pencil writing handed me by Dr. C.'s partner, which stated that "Dr. C." had been a student of

Dr. . . . . . , eight years professor in the Dental College. Dr. C. was entirely innocent of the blunder, and would be, I am well assured, as far from making a false statement to deceive the public, as the writer of the article above mentioned.

Yours, respectfully, . . . . . . . . . . . . . ., Editor, etc.

